# Open Systems, Quantum Probability, and Logic for Quantum-like Modeling in Biology, Cognition, and Decision-Making

**DOI:** 10.3390/e25060886

**Published:** 2023-06-01

**Authors:** Andrei Khrennikov

**Affiliations:** International Center for Mathematical Modeling in Physics and Cognitive Sciences, Linnaeus University, SE-351 95 Växjö, Sweden; andrei.Khrennikov@lnu.se

**Keywords:** open quantum systems, biology, cognition, decision-making, psychology, quantum logic and probability, quantum instruments

## Abstract

The aim of this review is to highlight the possibility of applying the mathematical formalism and methodology of quantum theory to model behavior of complex biosystems, from genomes and proteins to animals, humans, and ecological and social systems. Such models are known as quantum-like, and they should be distinguished from genuine quantum physical modeling of biological phenomena. One of the distinguishing features of quantum-like models is their applicability to macroscopic biosystems or, to be more precise, to information processing in them. Quantum-like modeling has its basis in quantum information theory, and it can be considered one of the fruits of the quantum information revolution. Since any isolated biosystem is dead, modeling of biological as well as mental processes should be based on the theory of open systems in its most general form—the theory of open quantum systems. In this review, we explain its applications to biology and cognition, especially theory of quantum instruments and the quantum master equation. We mention the possible interpretations of the basic entities of quantum-like models with special interest given to QBism, as it may be the most useful interpretation.

## 1. Introduction

The year 2022 was important for quantum information studies—Aspect, Clauser, and Zeilinger were awarded the Nobel Prize for experimental and theoretical studies on quantum foundations supporting quantum computing, cryptography, and teleportation. This is a good time to highlight the not so commonly known output of the quantum information revolution that is often called the second quantum revolution, namely, applications of quantum foundations and formalism outside of physics *quantum-like modeling* [1,2].

This is a review of *quantum-like modeling* and its applications, with an emphasis on the role of theory in open quantum systems. Such modeling is built on the methodology and the mathematical apparatus of quantum theory, and it is directed to applications in biology, cognition, psychology, decision-making, economics, finances, social and political sciences, and artificial intelligence. It is of essential importance to clarify that this approach can be explored for macroscopic systems, and the system’s size is not significant. The quantum-like framework is applicable on all scales, that is to say, from proteins and genes to animals, humans, and ecological and social systems. A crucial role is played by the character of information processing by a system, which matches with the laws of quantum information theory [3]. Systems are treated as information processors. Metaphorically, one may say that a system’s “hardware”, its physical and biological structures, are not so significant, but the system’s “software” plays the central role. We can speak about *quantum bioinformatics* [4], which should not be confused with *quantum biophysics* [5]. The latter studies the genuine quantum physical processes in biosystems, e.g., in cells.

This review cannot reflect all publications on quantum-like modeling. A Google search generates 213,000,000 results for “quantum-like modeling” in 0.47 s. (This is really surprising to me). In part, this review represents the content of a forthcoming book [6] by reflecting related references, especially regarding applications of the theory of open quantum systems to cognition and decision-making. The author (in cooperation with Accardi, Asano, Basieva, Ohya, and Tanaka) was a pioneer in employing quantum information and open systems outside of physics [7,8,9,10,11,12,13,14].

It is important to point out the immense influence of mathematics in physics, emphasized by many scientists and, in particular, by E. Wigner [15]. However, mathematical tools commonly used in theoretical and mathematical biology, cognition, and psychology are not as effective as in theoretical and mathematical physics. Gelfand pointed to *“the ineffectiveness of mathematics in biology”* [16]. From my point of view, Gelfand’s statement has to be reformulated, as one could instead speak about the ineffectiveness of mathematics that is *commonly used* in biology, cognition, and psychology. Presumably, someone has the intention to model micro-systems behavior, say of electrons, atoms, or photons, within classical analysis of functions defined in phase space, A=A(q,p). In this case, one would confront difficulties and soon would notice either the impossibility of such a description of quantum phenomena or at least its ineffectiveness. Personally, I stress the effectiveness of quantum description and do not highlight various no-go statements concerning the impossibilities in the classical description.

Physicists have explored a new branch of mathematics, a theory of operators in complex Hilbert space, in order to describe quantum phenomena in the effective way. In quantum physics, noncommutative operator calculus works very well. Similarly, one should search for novel mathematics techniques that are proper for biological and mental phenomena. The quantum-like approach utilizes the same mathematics employed in quantum physics, that is, noncommutative operator calculus in complex Hilbert space. Why is this so attractive for the applications discussed? Personally, I have mainly been driven by the particulars of quantum probability (QP) calculus that match mental phenomena very well. This point will be discussed later in detail. However, one can look at the deeper level as well. It is useful to discuss the basic problems in mathematical modeling of mental phenomena highlighted by experts in the field.

The central objective of this paper is to illuminate some directions in the development of quantum-like modeling [7,8,9,10,11,12,13,14,17,18,19,20,21,22,23,24,25,26,27,28,29,30,31,32,33,34,35,36,37,38,39,40,41,42,43,44,45,46,47,48,49,50,51,52,53,54,55,56,57,58,59,60,61,62,63,64,65,66,67,68,69,70,71,72,73,74,75,76,77,78,79,80,81,82,83,84,85,86,87,88,89,90,91,92,93,94,95,96,97,98,99,100,101,102,103,104,105,106,107,108]. First, we introduce the motivation for operating with quantum formalism and, particularly, probability outside of physics. Then, we compare classical and quantum probability (CP and QP) theories and set forth the principles of quantum-like modeling of decision-making. In particular, consideration of a special quantum-like model, “decision-making via decoherence” [9], leads to coupling with theory of open quantum systems [109]. The latter is discussed in more detail by highlighting its applications to the behavior of complex biosystems [110] (especially the problem of stability), cognition, the brain’s functioning, theories of consciousness, and emotional coloring of perceptions.

The theory of open quantum systems accommodates the formalism of quantum instruments [111,112,113,114]. This formalism realizes the most general quantum state updates. We stress that the combination of basic psychological effects, such as the question order effect (QOE) and the response replicability effect (RRE), encourages us to inquire into the possibility of proceeding using standard quantum measurement theory (with the representation of observables by Hermitian operators and the quantum state update via the projection postulate) [67]. We demonstrate that this psychological effect combination can be modeled with quantum instruments [94,95].

This article is written schematically, with a minimal introduction to quantum methodology and mathematical apparatus (see Appendix A and Appendix B).

## 2. Exploring Quantum Formalism and Methodology

For newcomers to the field of quantum-like modeling who are searching for its motivation, I can recommend two handbooks [57,115] on quantum models in social science and mathematical psychology, in particular, article [72] in the first book, and the preface of the second one. In article [72], I argue that functioning of biosystems should include cognition and, in particular, that unconscious–conscious interaction can be modeled within open quantum systems theory (see also [70]).

### 2.1. Edgar Allan Poe’s Reasoning in Favor of Quantum-like Modeling

The preface to the handbook on mathematical psychology [115] begins with the brilliant citation from a story written by Edgar Allan Poe (1845) entitled *The Purloined Letter*. In this story, the protagonist, Mr. C. Auguste Dupin, discussed the limits of the applicability of mathematics:

*“Mathematical axioms are not axioms of general truth. What is true of relation — of form and quantity — is often grossly false in regard to morals, for example. In this latter science it is very usually untrue that the aggregated parts are equal to the whole. […] two motives, each of a given value, have not, necessarily, a value when united, equal to the sum of their values apart.”*

One can be surprised by Poe’s doubts as to the applicability of the mathematics of the 19th century to moral phenomena (cf., with attempts of, for example, Freud to proceed with “classical mathematics”). He also expressed doubts about the validity of the value-additivity law. This is a very deep statement and, in quantum mathematics, it is formulated as “eigenvalues of the sum of operators C=A+B are not equal to the sums of the eigenvalues of the summands”, i.e., generally,

$$c_{i}\neq a_{i}+b_{i}.$$

In fact, the violation of the value-additivity law is the key point of von Neumann’s no-go theorem [116], which was the first statement about the impossibility of classical reduction of quantum theory. The authors of [115] also pointed out the noncommutativity effect in conjunctions,

A&B≠B&A.

This order effect is also naturally formalized in the quantum framework. In fact, these two effects, the value-nonadditivity and the order effects, are closely connected. In probabilistic terms, they are jointly expressed in the violation of the formula of total probability and the interference of probabilities [64,117,118].

The essential part of quantum-like modeling is devoted to the order effect [119]. Its QP-realization in decision-making has been accomplished in article [120] (see also [121]).

Finally, we remark that someone can be skeptical and ask whether some mental features are at all representable with mathematical models. To stay with the above example, one can ask whether morals can be given comprehensive mathematical treatment at all. Such doubts are rather common for neuro-physiologists and brain scientists. My personal belief is that mathematical modeling can clarify a lot about the brain’s information processing. For the moment, it is not clear at all which mathematical model might be successful. The quantum-like approach is promising, and it has been successful for modeling some cognitive tasks and psychological effects. However, it is too early to claim that this approach is really adequate for explaining the mental phenomena. This discussion will be continued in Section 6.5, which is devoted to quantum-like modeling of unconscious–conscious interactions.

### 2.2. Mathematical Models of Mental Phenomena: Why Quantum?

We remark that in [115] the discussion is not coupled to quantum-like modeling: the authors searched for novel mathematical tools for psychology, but their considerations call for an appeal to quantum formalism. The main message [115] was that a variety of mathematical methods could be explored to solve the problems mentioned by Edgar Allan Poe, with which I completely agree. Quantum formalism should not be treated as pretending to be the unique mathematical tool for modeling of mental phenomena. A while ago, in response to the developments of using quantum formalism outside of quantum mechanics, the eminent quantum physicist Anton Zeilinger (Nobel Laureate 2022) told me,

*“Why should it be precisely the quantum mechanics formalism? Maybe its generalization would be more adequate for mathemtical modeling of mental phenomena …”*

He is correct that, for the moment, despite its tremendous success, quantum-like modeling is still at the testing stage. Perhaps one day, a new and more advanced mathematical formalism will be suggested for modeling in cognition, psychology, and decision-making.

### 2.3. Simplicity, Elegance, and Generality

From my viewpoint, quantum formalism is very successful due to its simplicity. The reader may be surprised: “Simplicity? But quantum theory is mysterious and very complicated!” One can immediately recall the famous statement commonly attributed to Richard Feynman,

*“I think I can safely say that nobody understands quantum mechanics.”*

Here, “understanding” is related to the interpretation problem of quantum mechanics; its formalism, however, is very simple: it is linear algebra. In the form of quantum information theory [3] that is the most useful for applications, including quantum engineering, linear state spaces are finite dimensional. Therefore, this is the matrix calculus in $H=C^{n}.$ Linear evolution is very rapid, and this is the advantage of quantum-like representation of mental states and their corresponding linear processing.

We also emphasize the generality of mathematical modeling based on quantum formalism: the same formalism and methodology cover a variety of biological and mental processes.

### 2.4. The Quantum-like Model as a Linear Approximation of Nonlinear Biological Processes?

In engineering, linear models often appear as approximations of essentially more complex nonlinear ones. Hence, the quantum-like representation of biological and mental phenomena might just be an approximation of more complex nonlinear processes in living systems. This viewpoint on quantum theory can be questioned. One of the reviewers remarked,

*Quantum theory is often presented as more general than classical theory. How does this square with the idea that quantum theory might be just a linearization of a complex, non-linear but classical theory? Is this relevant for understanding why quantum theories of mental processes might be good models for the (non-linear) system of the brain?*

The linearization viewpoint on quantum theory is supported by a few theoretical models of emergence of quantum theory from nonlinear classical theories.

First, I point to my own studies devoted to *prequantum classical statistical field theory* (PCSFT) [122]. Within PCSFT, quantum mechanical formalism is derived from nonlinear classical field theory. Quantum formalism is treated as a machinery for approximate calculation of probabilities and averages. In particular, the Born rule appears as an approximate rule. Linear Schrödinger dynamics is also an approximation of nonlinear subquantum dynamics. PCSFT has been used in cognitive modeling (see [106] for details).

One can also refer to numerous attempts to modify quantum theory and create a more general quantum theory with nonlinear dynamic equations. Einstein was the pioneer of such studies, and his attitude was expressed very clearly in the book by Einstein and Infeld [123]; Einstein worked on the creation of such a theory the last 20 years of his life, but without success. Later, Białynicki-Birula (who was a graduate student of Infeld) tried to develop the ideas of Einstein and Infeld, and he proceeded rather far in construction of nonlinear quantum mechanics [124]. The main problem of the Einstein–Infeld–Białynicki-Birula approach is overly direct coupling to the standard mechanics. The most difficult problem of such nonlinear quantum theories is the formulation of a nonlinear analog of the superposition principle. Unfortunately, they were not successful in solving this problem. In contrast, coupling between PCSFT and quantum mechanics is more difficult and provides more consistent interrelation between them.

## 3. Classical vs. Quantum Probability

This short section presents the motivation for employing quantum probability (QP) instead of classical probability (CP) for mathematical modeling in cognitive psychology and decision-making. We refer to the works of Kahneman (the Nobel Prize laureate in economics) and Tversky (the most cited psychologist) [125,126,127,128,129], who pointed out that using CP as the basis of decision theory leads to inconsistencies and paradoxes (such as the Allais [130] and Ellsberg [131] paradoxes; see also Erev et al. [132]).

Such a motivation is not grounded in foundational principles. Here, we proceed in parallel with quantum physics, a field created to resolve inconsistencies between classical electrodynamics and experimental data for black-body radiation.

In cognitive psychology and decision-making, experiments on irrational behavior and probability fallacies (including experiments demonstrating the basic paradoxes of Allais, Ellsberg, and Machina) have generated a lot of statistical data that do not match, at least straightforwardly, with the main CP laws [65]. These data were analyzed and modeled within the QP framework. We mention some work along these lines [14,18,39,41,53,65,101,104]. This is just a sample from a series of publications on QP-structuring of statistical data collected over the last 50–60 years in psychology, cognition, decision-making, and economics. A detailed analysis of these publications charaterized by a variety of quantum techniques is a topic for a separate review.

### 3.1. Contextuality: Physical vs. Mental

To support the use of QP, we highlight *the quantum-like paradigm* [2] by which context-sensitive systems, including humans, process information in the form of superpositions, i.e., without ambiguity resolution. Such processing can be effectively described by quantum formalism. Once again, we appeal to effectiveness of the QP description, not to the impossibility of exploring CP. Generally proving various no-go statements is counterproductive, in both physics and decision-making. If one is able to describe physical or mental phenomenon with CP vs. QP, and if this description is effective, then one can proceed with CP vs. QP.

QP is a contextual probabilistic formalism [64]. Its main power is in operating within incompatible experimental contexts, with observables for which the joint probability distribution is not well-defined. Contextuality is a hot topic in quantum information theory. Quantitative methods for estimation of the degree of contextuality are formed with the aid of Bell-type inequalities. This topic is novel for psychology, cognition, and decision-making. To generate statistical data, new experiments should be performed. Outputs of such experiments can be found in articles [133,134,135,136,137,138] demonstrating violation Bell-type inequalities (treated as non-contextual inequalities). This line of research is very promising. It provides a fresh view of mental contextuality and new mathematical methods for its estimation. We remark that inter-contextual experimenting is not typical for mental studies.

#### Mental Hysteresis

QP-stimulated research on mental contextuality has led to the experimental discovery of *mental hysteresis* [139,140,141], which has some similarity with physical hysteresis, e.g., for ferromagnetic materials. In an optical illusion experiment for an ambiguous figure, a figure was cyclically rotated, and the probabilities of its recognition were determined for each angle. These probabilities form the hysteresis curve with respect to the angle axis. Up to now, only one experiment on this mental phenomenon has been performed [139,140,141] (at the Tokyo University of Science). Unfortunately, the interest in this study of decision-making and psychology was negligible. I am thankful to Anton Zelinger, who emphasized the possible future impact of the discovery of mental hysteresis. Of course, his comment (at one of the Växjö conferences on quantum information, probability, and foundations) expressed the opinion of a physicist, with an opinion based on his physical intuition.

Finally, we point out that QP and the theory of open quantum systems have been applied to genetics and molecular biology [13]. Cells as well as DNA and RNA molecules can be treated as decision-makers. In particular, we studied gene expression and represented the process of consumption of glucose and lactose by a cell as a quantum-like interference [10]. Moreover, new experiments on populations of *E. coli* bacteria were performed at the molecular biology lab of the Tokyo University of Science by Tanaka under supervision of Yamato, a professor of genetics.

## 4. Quantum Formalism for Decision-making

We recount the fundamentals of the quantum-like modeling of decision-making, e.g., [39,41,61,65,100]. The basic scheme explores the standard quantum measurement theory. The basic components are represented as follows:

- Questions, problems, and tasks as quantum observables, Hermitian operators;
- Belief or mental states of decision-makers by quantum states normalized vectors in a complex Hilbert space or general density operators;
- The quantum state updated via the projection postulate.

From the very beginning, we have highlighted that, in applications of the quantum measurement theory to cognition and general biology, a crucial role is played by finding the proper formalization of the state update resulting from decision-making. The projection state update is the simplest one, and it cannot cover all cognitive phenomena. More general state updates are explored in quantum information theory; they are formalized within quantum instruments theory (Section 7).

We also mention a special quantum-like model, *“decision-making via decoherence”*, coupled with open quantum systems theory (Section 6.4).

The problem of the belief-state interpretation can be discussed by paying attention to the diversity of possible interpretations [142], which is one of the problems of quantum foundations. We recount the basic quantum state interpretations, namely, individual and statistical. We also mention QBism [143,144,145,146,147,148,149,150] as perhaps the most useful framework for quantum-like decision-making.

I have a rather strange relationship with QBism and its creators. In fact, QBism has shown up prominently at the Växjö conferences on quantum foundations since the year 2000 [143,144,151]. Initially, I actively struggled against QBism [152] since, for me, the use of subjective probability in quantum physics and in statistical physics in general is nonsense. In particular, I actively disrupted Christopher Fuchs during his talks in Växjö by trying to explain him that probability cannot be assigned to individual physical events. However, by being more involved in quantum-like modeling for decision-making, I began to treat QBism as perhaps the best interpretation for QP quantum-like decision-making [153,154].

This is a good place to point out the applications of QBism to decision-making in geology, specifically regarding the project on determination of the utility of the perspective for intelligent petroleum reservoir characterization, monitoring, and management [155,156]

## 5. Quantum and Classical Logic of Thought

We start this section with the remark that Boole designed classical (Boolean) logic for “investigation of the laws of thought” [157].

Although I have put so much effort into the justification of quantum-like modeling through QP analysis and especially its contextual nature, I have slowly started to understand that the seed of cognition’s quantumness (not only of humans, but also other biosystems) is in the logic structure of information processing (see, e.g., articles on quantum-like modeling of the problem of common knowledge and violation of the Aumann theorem [158,159] and on the impossibility of agreeing to disagree [108,160]).

Quantum logic corresponds to the linear representation of information. The basic law distinguishing classical (Boolean) and quantum logic is the distributivity law, which is violated in quantum logic (see article [161] for details). We now briefly recall the basics of quantum logic [162,163] (cf., classical logic [157,164]).

Logical operations are defined on subspaces of complex Hilbert space H or equivalently on the set of orthogonal projectors P(H). Subspaces (projections) are representations of propositions (events). Let *P* be projection, and let $L_{P}$ be its image, $L_{P}=PH.$ For a subspace L, $P_{L}$ is the projection on *L*. Denote the projection onto the orthogonal complement to the subspace $L_{P}$ by the symbol $\bar{P},$ i.e., $H=L_{P}⊕L_{\bar{P}}.$

Negation of the proposition *P* is represented by $\bar{P}.$ The operations of conjunction ∧ and disjunction ∨ are defined as follows.

Let *P* and *Q* be orthogonal projections representing some propositions. The conjunction–proposition (event) P∧Q is defined as the projector on the intersection of subspaces $L_{P}$ and $L_{Q},$ i.e., $L_{P\wedge Q}=L_{P}\cap L_{Q}.$ We remark that this operation is well-defined even for noncommuting projectors, i.e., incompatible quantum observables. Moreover, it is commutative:

(1) P∧Q=Q∧P.

The same can be said about the operation of disjunction. Here, subspace $L_{P\vee Q}$ is defined as the subspace generated by the union of subspaces $L_{P}$ and $L_{Q},$ i.e., P∨Q is a projector on this subspace. This operation is also well-defined for non-commuting projectors and, moreover, it is commutative:

(2) P∨Q=Q∨P.

Thus, quantum logic is commutative logic. This fact is never highlighted. Thus, in quantum reasoning, noncommutativity is not present at the level of the basic operations of quantum logic, conjunction, and disjunction. In light of this fact, the following natural question arises:

*What is the logical meaning of noncommutativity of quantum operators?*

We can recall that noncommutativity is commonly considered the basic mathematical feature of quantum theory. Hence, it should also play a crucial role at the level of quantum logic. The answer is rather unexpected, and it is given by Theorem 1 below.

As one knows, classical Boolean logic is distributive [157,164], i.e., for any three propositions (events) X,Y,Z, e.g., represented by subsets of some set,

(3) X∧(Y∨Z)=(X∧Y)∨(X∧Z).

**Theorem 1.**  *[161] Let P,Q,R be projections. They are pairwise commutative if and only if the distributivity law (3) holds for $X,Y,Z=P,Q,R,\bar{P},\bar{Q},\bar{R}.$*

Thus, noncommutativity encodes non-distributivity of quantum logic! Hence, the existence of incompatible quantum observables, i.e., represented by noncommuting operators, is equivalent to non-transitivity of logical relations between quantum propositions. In particular, Bohr’s complementarity principle [165,166,167,168,169] reflects this logical structure.

This statement is especially important for quantum-like modeling of cognition. The existence of incompatible propositions or questions is a consequence of non-distributivity of logic used by a quantum reasoner.

*Are humans classical or quantum reasoners?*

The answer to the above depends on the context of information processing. In some situations, humans use distributive Boolean logic, but in other situations, they violate distributivity law, and quantum logic can be employed to mathematically describe the latter form of reasoning.

At first glance, it seems to be impossible to characterize distributive vs. non-distributive (classical vs. non-classical) reasoning in an experimentally testable way. However, such a characterization was obtained in [161] based on testing of the response replicability effect (RRE).

The notion of *response replicability* plays an important role in quantum physics. This property of observations is also a common feature of human behavior. Suppose that Alice is asked some question *A*, and she replies, e.g., “yes”. If, immediately after answering, she is asked this question again, then she replies “yes” with a probability of 1. This is called *A−A response replicability.* In probabilistic terms, this notion is formalized as

(4) $$Pr\left(A=x,A=x^{\prime }\right)=0,\;x\neq x^{\prime }.$$

A−A RRE is valid for a classical observable represented by a random variable. Let P=(Ω,F,P) be a Kolmogorov probability space (see Appendix A), and let *A* be a discrete random variable. Then,

(5) $$Pr\left(A=x,A=x^{\prime }|P\right)\equiv P\left(\omega \in \Omega :A\left(\omega \right)=x,A\left(\omega \right)=x^{\prime }\right)=0,\;x\neq x^{\prime }.$$

A−A RRE is also valid for a quantum observable represented by Hermitian operator A. Let $\left(E_{A}\left(x\right)\right)$ be its spectral family of projections corresponding to the eigenvalues (we consider only operators with discrete spectra), i.e.,

$$A=\sum_{x}xE_{A}\left(x\right).$$

Then, for a pure state ψ,

(6) $$Pr\left(A=x,A=x^{\prime }|\psi \right)\equiv \left|\right|E_{A}\left(x\right)E_{A}\left(x^{\prime }\right)\psi {\left|\right|}^{2}=0,\;x\neq x^{\prime }.$$

This is the distinguishing feature of quantum observables of the projection type.

Human decision-making commonly involves another property—*A−B−A response replicability.* Suppose that after answering the *A*-question with the “yes”-answer, Alice is asked another question B. She replies with some answer, and then she is asked *A* again. In social opinion polls and other natural decision-making experiments, Alice definitely repeats her original answer to A, “yes”. This is A−B−A response replicability. In probabilistic terms, this notion is formalized as

(7) Pr(A=x,B=y,A=x)=Pr(A=x,B=y).

A−B−A RRE is valid for classical observables represented by random variables. Let A,B be discrete random variables. Then,

(8) Pr(A=x,B=y,A=x|P)≡P(ω∈Ω:A(ω)=x,B(ω)=y,A(ω)=x)=Pr(A=x,B=y|P).

A−B−A RRE is also valid for quantum observables represented by commuting Hermitian operators A,B. Let $\left(E_{A}\left(x\right)\right)$ and $\left(E_{B}\left(y\right)\right)$ be their spectral families—projections corresponding to the eigenvalues. Then, for a pure state ψ,

(9) $$Pr\left(A=x,B=y,A=x|\psi \right)\equiv \left|\right|E_{A}\left(x\right)E_{B}\left(y\right)E_{A}\left(x\right)\psi {\left|\right|}^{2}=\;\left|\right|E_{A}\left(x\right)E_{B}\left(y\right)\psi {\left|\right|}^{2}=Pr\left(A=x,B=y|\psi \right).$$

Since commutativity implies the existence of the joint probability distribution, (8) implies (9).

The combination of A−A with A−B−A and B−A−B response replicability is called *the response replicability effect* (RRE). This effect holds for classical observables, random variables, and compatible quantum observables of the projection type.

**Theorem 2.**  *[161] The projection observables P and Q show RRE in state ψ if and only if the distributive law holds for this state, i.e.,*

(10) [X∧(Y∨Z)]ψ=[(X∧Y)∨(X∧Z)]ψ,

*for $X,Y,Z=P,Q,\bar{P},\bar{Q}.$*

As was shown in [161], RRE can be checked experimentally. For the moment, only one experimental test has been done [170], and its design and methodology were questioned by a few researchers; see comments on the PLOS One webpage for this article. Reference [161] could possibly attract the attention of experimenters to RRE as a test for the non-classicality of human logic.

### 5.1. Quantum vs. Quantum-like Cognition

We emphasize that quantum-like modeling of cognition should be sharply distinguished from quantum brain studies (see, e.g., [171,172,173,174,175,176,177,178,179,180]) attempting to reduce information processing by cognitive systems, including “generation of consciousness”, to quantum physical effects in the brain. However, we do not criticize the quantum brain project, even though its difficulties are well known; e.g., the brain is too hot and big, and the scales of neurons operating in the brain are too rough compared to quantum physical scales.

In quantum-like modeling, it is simply not important whether the genuine quantum physical processes in brain cells contribute to cognition or not. Generally, quantum-like modeling is performed on the meta-level of cognition; it does not concern the biophysical processes in neurons. In this framework, a biosystem—specifically, the brain—is treated as a *black box* whose information processing cannot be described by *classical probability* [181] (CP) and, hence, by classical information theory. Non-classical probability and information theories are in demand. In particular, in decision-making, exploring CP leads to various paradoxes that are typically coupled to the irrational behavior of humans. My suggestion [2] was to employ quantum probability (QP) and quantum information theories instead of classical ones. Why should special quantum theory be involved? This is a complex problem.

### 5.2. Classical, Quantum, or More General Probability Theories?

There exist a plethora of other models that are different than CP and QP. The use of QP in, e.g., decision-making was not derived from basic principles of cognition and psychology. Commonly, QP is used pragmatically, to resolve the basic paradoxes of classical decision theory and to apply a general probabilistic framework to decision-making in all areas of humanities and economics, as well as in biology. There is no a priori reason to hope that QP will cover all problems that arise in decision-making. One might find paradoxes even in QP-based decision theory. Perhaps other probabilistic models different from both CP and QP should be employed.

Surprisingly, physicists have the same problem. In contrast to relativity theory, QM was not derived from natural physical principles (see Zeilinger [182] for a discussion of this problem). There is no reason to expect that all experiments in the micro-world would match QP constraints.

In physics, one typically debates CP vs. QP and classical vs. quantum physics. However, one can test whether the physics of microsystems violate QP laws, i.e., whether electrons and photons can behave exotically even from a QP viewpoint. The corresponding test is given by the *Sorkin equality* [183] for the three-slit experiment. It is really surprising that two- and three-slit experiments have such different probabilistic structures. The three-slit experiment was done by the Weihs group (Austria). They did not find deviations from QP, and the Sorkin inequality was not violated [184,185]. Similar experiments can be done for decision-making by humans by using the theoretical formalism of the Sorkin inequality in terms of quantum probabilities [186].

## 6. Biosystems as Open Quantum-like Systems

Any living biosystem is an open system and, to analyze its behavior, it is reasonable to take advantage of the open quantum systems theory, whether or not the biosystems are acknowledged as information processors and the open quantum systems theory is treated as part of the quantum information theory. The latter is the most general information theory, with classical information theory as a particular case. Thus, the open quantum systems part of quantum-like modeling concerns information processing in complex biosystems interacting with their environments. From the information point of view, even cells or proteins are very complex systems.

The challenging problem of mathematical formalization of the unconsciousness–consciousness interrelation can also be handled by the open quantum systems theory [70,82]. Consciousness plays the role of an apparatus performing measurements over unconsciousness. This formalism matches well with the Higher Order Theory of Consciousness [187,188,189,190,191]. It is used to mathematically model the emotional coloring of conscious experiences. Such coloring is framed as contextualization. Therefore, the theory of emotions is coupled with the hot topic of contextuality in quantum foundations. Finally, we discuss the Bell-type experiments [192,193,194,195] for emotional coloring [82] (see also [133,134,135,136,137,138] for such experiments in cognition and decision-making).

### 6.1. What Is Life?

Treating biosystems as quantum-like information processors can explain order stability in them, i.e., present the quantum-like formalization of Schrödinger’s speculations in his well-known book *“What is life?"* [196] (see [197]). Schrödinger stressed that order stability is one of the characteristic features of biosystems. Entropy can be used as a quantitative measure of order. He also noted that, in physical systems, entropy has the tendency to increase (the Second Law of Thermodynamics for isolated classical systems and dissipation in open classical and quantum systems). In contrast, biosystems exceed this tendency. Schrödinger asked: “How?” Quantum information and open systems theory may give the answer to this fundamental question of modern science.

In [197], the process of biosystems’ adaptation to the surrounding environment is described by the *Gorini–Kossakowski–Sudarshan–Lindblad equation* [110], where the von Neumann and linear quantum entropies are employed as measures of the disorder degree. This equation describes Markovian evolution, so we work with quantum Markov dynamics. Markovianity possesses the strong constraint on the class of mental state dynamics. The description of information processing in biosystems with quantum non-Markovian dynamics is more promising, but at the same time it is more complicated.

We highlight the role of a special class of quantum dynamics that generates a camel-like shape for quantum entropies. The camel’s hump represents:

- (a) The entropy increase in the process of the initial adaptation to the environment;
- (b) The entropy decrease at the post-adaptation stage of the dynamics.

Our analysis [197] is based on a numerical simulation, and the analytical description of such a class of quantum dynamics is necessary.

### 6.2. Order Stability in Complex Biosystems in Spite of Instability in Subsystems

Once again, the theory of open quantum systems is used (see [198]) in attempting to bring more clarification to the question “What is life?” We consider a complex biosystem *S* composed of many subsystems, such as proteins, cells, or neural networks in the brain, i.e., $S=(S_{i}).$ We study the following problem:

*Whether the composed system S can preserve the “global order” in the situation of increase in local disorder and whether S can preserve its entropy while some subsystems $S_{i}$ increase their entropies.*

It is shown that, within quantum information theory, the answer is positive [198]. Entanglement of the subsystems’ states plays a crucial role. In the absence of entanglement, the increase in local disorder generates the increase in disorder in the compound system *S* (as in the classical regime).

### 6.3. Modeling of Brain Functioning: From Electrochemical to Quantum Information States

As an application of the open quantum systems theory to cognition, we suggest a quantum-like model of the brain’s functioning (see [70,76,82]). In this model, the general approach of quantum-like modeling—beginning directly with the quantum information representation of the biosystems’ states—is broken. We start by considering the electrochemical states of neurons encoded in action potentials. Such states generate the brain’s mental states, which are processed with open quantum systems dynamics.

The model does not refer to the genuine quantum physical processes in the brain. Hence, it does not suffer from the well-known problem of matching the quantum and neural scales, temporal, spatial, or temperature (cf., [171,172,173,174,175,176,177,178,179,180]). In this model, uncertainty generated by the action potential of a neuron is represented as quantum-like superposition of the basic mental states corresponding to some neural code, e.g., quiescent/firing neural code.

Mathematically, the neurons’ state space is described as a complex Hilbert space of quantum information states. The state of a neural network is presented in the tensor product of single-neuron state spaces.

The brain’s mental functions perform self-measurements by extracting concrete answers to questions from the quantum information states. This extraction is modeled in the framework of the theory of open quantum systems.

The notion of self-measurement (or self-observation) as used in this paper can be associated with the notion of representation employed by neuroscientists. This is not based on the idea of “someone observing something” (cf., Dennett’s critique of the homunculus [199]).

The dynamics of the state of mental function *F* are described by the quantum dynamical equation. Its stationary states represent classical statistical mixtures of possible outputs (decisions) of mental function F. A stationary state $\rho_{\mathrm{stationary}}$ determines the probabilities of possible outcomes. How does *F* select one concrete outcome? A possible model is based on a classical random generator coupled to *F*, generating its outcomes with probabilities encoded in $\rho_{\mathrm{stationary}}$ (see [9,10,11,12,13,14] for details). In this way, one can escape appealing to the state collapse.

### 6.4. Decision-making via Decoherence

The above scheme of resolution of uncertainty through interaction with the environment is known as quantum dynamical decision-making or *decision via decoherence* [9,10,11,12,13,14] (see also [200] on the experimental study of eye tracking in the process of decision-making and its modeling with the Gorini–Kossakowski–Sudarshan–Lindblad equation [110]). Here, the experimentally observed stabilization in eye tracking matches perfectly with stabilization of the solution to this equation. One of specialties of this model is in employing three-dimensional space of mental states. The equation is phenomenological, i.e., it is not derived from neurophysiology beyond eye moving in the process of decision-making. We hope that this study will stimulate further cooperation for finding the physical and physiological signatures of the mental state stabilization in the process of decision-making. It would also be interesting to perform new experiments with a design similar to the experiment in [200] and check whether the same phenomenological equation as in [200] can be used.

The most promising realization of this scheme is the “differentiation” model [201], by which the mental state experiences step-by-step state transitions under the influence of surrounding electrochemical environmental factors. The differentiation leads to stabilization of the biosystem’s state. This model is applicable for all biological and social scales, from cells to ecological and social systems.

Decoherence is a deep foundational notion. Heuristically, it can be interpreted as the loss of quantumness, transition from QP to CP, and washing out of interference of probabilities (see, e.g., Zurek [202]). It is quantified with linear entropy, or the measure of a state’s purity. In quantum information theory, typically decoherence is considered a negative factor disturbing information processing. In quantum dynamical decision-making, decoherence plays a constructive role as a generator of decisions.

### 6.5. Consciousness as Observer on Unconsciousness: Quantum Formalization

The open quantum systems theory is also used for mathematical formalization of the consciousness–unconsciousness interaction, as well as the information exchange between them. Consciousness plays the role of a measurement device, as it performs observations over the states of unconsciousness. These observations can be interpreted as the brain’s self-observations. Therefore, a human’s thoughts and decisions are generated in the complex process of interaction between unconscious and conscious states. From the viewpoint of quantum foundations, we use Bohr’s interpretation of the outcomes of quantum measurements as generated in the complex process of interaction between a system and measurement apparatus [165,166,167,168,169]. In particular, these outcomes are not objective properties of a system that could be associated with it before measurement. This is a good place to present the following widely cited statement of Bohr (1949) [165]:

*This crucial point … implies the impossibility of any sharp separation between the behaviour of atomic objects and the interaction with the measuring instruments which serve to define the conditions under which the phenomena appear. In fact, the individuality of the typical quantum effects finds its proper expression in the circumstance that any attempt of subdividing the phenomena will demand a change in the experimental arrangement introducing new possibilities of interaction between objects and measuring instruments which in principle cannot be controlled. Consequently, evidence obtained under different experimental conditions cannot be comprehended within a single picture, but must be regarded as complementary in the sense that only the totality of the phenomena exhausts the possible information about the objects.*

We want to connect quantum foundations and especially this specific statement by Bohr with consciousness studies. As is well known, there are two basic competing theoretical frameworks for consciousness (see [203] for a more systematic classification):

- First Order Theory of Consciousness [204,205,206,207,208];
- Higher Order Theory of Consciousness [171,172,173,174,175,176,177,178,179,180,181,182,183,184,185,186,187,188,189,191,199,203,209,210,211].

These theories can be characterized according to [191] as follows.

First-order theorists, such as Block, argue that processing related to a stimulus is all that is needed for there to be phenomenal consciousness of that stimulus [204,205,206,207,208]. Conscious states, in this view, are states that make us aware of the external environment. Additional processes, such as attention, working memory, and metacognition, simply allow cognitive access to and introspection about the first-order state. In the case of visual stimuli, the first-order representation underlying phenomenal consciousness is usually said to involve the visual cortex, particularly the secondary rather than the primary visual cortex. Cortical circuits, especially those involving the prefrontal and parietal cortex, simply make possible cognitive (introspective) access to the phenomenal experience occurring in the visual cortex.

In contrast, David Rosenthal and other higher-order theorists argue that a first-order state resulting from stimulus-processing alone is not enough to make possible the conscious experience of a stimulus. In addition to having a representation of the external stimulus, one also must be aware of this stimulus representation. This is made possible by a HOR (higher order representation), which makes the first-order state conscious. In other words, consciousness exists by virtue of the relation between the first- and higher-order states. Cognitive processes, such as attention, working memory, and metacognition, are key to the conscious experience of the first-order state. In neural terms, the areas of the GNC, such as the prefrontal and parietal cortex, make conscious the sensory information represented in the secondary visual cortex.

The Higher Order Theory sharply distinguishes between unconscious and conscious mental processing in the brain. Cognition is made conscious via a higher-order *observation* of the first-order processing. In QM, an observation is not simply non-disturbative inspection of the state of a quantum system. This is a complex process of interaction between a system and apparatus used for measurement. The latter statement is interpretation dependent. In the physical state, collapse theory observation is reduced to instantaneous state collapse.

Bohr’s viewpoint on quantum measurement matches the Higher Order Theory of Consciousness. A conscious experience is not simply introspection of the unconscious state, but rather a complex interaction process modifying the state of the compound system unconscious–conscious.

In the quantum-like model, unconsciousness and consciousness are two information processors, denoted by the symbols UC and C. The corresponding state spaces, complex Hilbert spaces, are denoted by the symbols $H_{\mathrm{UC}}$ and $H_{C}.$ Their tensor product $H=H_{\mathrm{UC}}⊗H_{C}$ is the state space of a compound system S=(UC,C). C performs measurements of various mental observables on the states of UC.

Consider a mental observable *A* (task, question) described mathematically by a Hermitian operator denoted by the same symbol A. Its measurement is based on interaction between the states of unconsciousness and consciousness, described mathematically by density operators ρ and Σ, acting in $H_{\mathrm{UC}}$ and $H_{C}$, respectively. The interaction is descried as unitary operator U:H→H. Here $U\equiv U_{A},$ i.e., interaction depends on the observable under measurement. This is a good place to remark that “quantum interaction” should not be identified with a classical force-like interaction. In the quantum information framework, *U* represents the information exchange between the states ρ and Σ.

When C starts the measurement process over UC, the state *R* of the compound system *S* (“brain’s state”) is separable, i.e., R=ρ⊗Σ. Then, the interaction operator *U* generates the entangled state $R_{U}=URU^{★}.$ The process of observation is probabilistic, and the output A=x is generated with its probability determined by the quantum instrument $I_{A}\left(x\right)$ (see Appendix B for an introduction to quantum instruments). The change of the state ρ of the system *S* caused by the measurement for the outcome A=x is represented with the aid of the map $I_{A}\left(x\right)$ in the space of operators defined as

(11) $$I_{A}\left(x\right)\rho ={\mathrm{Tr}}_{H_{C}}\left[\left(I⊗E_{A}\left(x\right)\right)U\left(\rho ⊗\sigma \right)U^{★}\right],$$

where $A=\sum_{x}xE_{A}\left(x\right)$ is the spectral decomposition of the Hermitian operator A. This is the special application of the so-called indirect measurement scheme (see [94] for a simple introduction). The process of conscious observation of unconscious states is probabilistic. P(A=x|ρ) is determined by Formula (A2); see Appendix B.

The main message is that one can try to formalize unconsciousness–consciousness interaction and generation of conscious experiences within the quantum information and open systems framework. Thus, such still-mysterious spheres of mental information processing can be embedded into quantum measurement theory. The latter is characterized by operating with *incompatible observables.*

In the quantum-like model, consciousness can obtain answers to incompatible questions. Such questions cannot be combined consistently. Nevertheless, consciousness of the quantum reasoner can handle them separately. Since incompatibility means the absence of the common probabilistic picture, the quantum reasoner does not try to construct the joint probability space for such questions. Construction of the classical probabilistic representation consumes time and computational resources. Therefore, the use of the quantum-like representation can save resources.

In [70], this model was employed to model the process of transformation of sensation into perceptions. This is the quantum realization of the von Helmholtz theory of sensation-perception [212]. According to von Helmholtz [212], perceptions are not simply copies of sensations, nor “impressions like the imprint of a key on wax”; they result from the complex processing of signals coming from the external environment, including unconscious cognitive processing. Creation of a perception is described in [213] as follows:

*Sensory information undergoes extensive associative elaboration and attentional modulation as it becomes incorporated into the texture of cognition. This process occurs along a core synaptic hierarchy which includes the primary sensory, upstream unimodal, downstream unimodal, heteromodal, paralimbic and limbic zones of the cerebral cortex.*

It is important to stress that the above presentation is not about “understanding” or “explanation” of unconsciousness and consciousness and their interaction. We are discussing mathematical modeling. In fact, physics is not about “understanding” or “explanation” either; it is also about mathematical modeling. I like to illustrate the situation in physics by referring to the theory of the electromagnetic field. Its basic entities, the electric and magnetic fields, E(t,x) and B(t,x), are just mathematical variables. They cannot be measured (only energy can be measured; i.e., the quantity $\int_{O}{\left(|E\left(t,x\right)\right|}^{2}+{\left|B\left(t,x\right)\right|}^{2})dxdt,$ where *O* is a domain in space-time). Before Einstein’s theory of relativity rejected ether, one could imagine the fields as the waves in the ether ocean. However, in modern physics, even such a heuristic picture is impossible. QM is even worse, as it can only predict the probabilities of observations and mathematically model the process of the state evolution. A quantum state |ψ〉 is also a mathematical quantity used for prediction of probabilities. In this sense, the aim of quantum-like modeling of unconsciousness–consciousness is to mathematically structure conscious experiences.

### 6.6. Emotional Coloring of Conscious Experiences

We start by citing LeDoux and Brown [191]:

*Emotion schema are learned in childhood and used to categorize situations as one goes through life. As one becomes more emotionally experienced, the states become more differentiated: fright comes to be distinguished from startle, panic, dread, and anxiety.*

This statement can be interpreted as follows: each emotion-generation scheme is crystallized on the basis of life-contexts. Emotions are employed markers of contexts corresponding to the surrounding physical and mental environment.

As was emphasized by Ekman [214], *emotions represent adaptive reactions to environmental challenges; they are a result of human evolution; they provide optimal (from the viewpoint of computational resources) solutions to ancient and recurring problems that faced our ancestors.*

The unconscious–conscious framework can be explored for quantum-like modeling of interconnected dynamics of perceptions and emotions [82]. More generally, this framework describes the emotional coloring process for a variety of conscious experiences, including decision-making. Two classes of observables are considered: perceptions and emotions. These observables are represented by Hermitian operators acting in the corresponding unconscious state spaces (or, more generally, by projection valued measures or PVMs). The total unconscious state space is their tensor product. Emotional coloring is structured within quantum contextuality formalism: emotional observables determine contexts. Such contextualization reduces degeneration of spectra for observables representing conscious experiences such as, for instance, perceptions or decision-making.

Reference [82] concludes with an experimental test of contextual emotional coloring of conscious experiences (cf., [133,134,135,136,137,138]), namely, on the violation of the CHSH inequality—the special Bell inequality associated with the names of Clauser, Horne, Shimony, and Holt [195]. Undertaking emotion-contextuality experiments such as this could serve as a step towards the experimental justification of the quantum-like model of emotional contextualization of conscious experiences.

## 7. Quantum Instruments in Physics, Psychology, and Decision-making

One of the specialties of my research over the last few years is exploring quantum instruments [111,112,113,114] in applications for psychology and decision-making, starting with article [70] devoted to modeling unconscious–conscious interaction. Quantum instruments are the basic tools of the modern theory of quantum measurements and open quantum systems. They describe the probability distributions of measurements’ outcomes and the quantum state transformations generated by measurements’ feedback. Thus, a quantum instrument describes both probability and its update via quantum state update.

Such updates are not reduced to ones based on the projections, i.e., given by the Lüders projection postulate. Rather, more general state space transformations are also needed, both in quantum physics, especially quantum information theory, and in quantum-like modeling, e.g., in decision-making and psychology.

Instruments can represent quantum observables by a *positive operator valued measure* (POVM), or generalized quantum observables. Typically, POVMs are considered the basic entities of the modern theory of quantum measurements, especially in quantum information theory. However, POVMs are just byproducts of quantum instruments. POVMs do not uniquely determine a state transformation coupled to a measurement’s feedback on a system’s state.

In physics, quantum instruments and, in particular, POVMs associated with them were introduced at the advanced stage of quantum theory’s development. However, modeling of cognition and decision-making should be based on quantum instruments even for basic psychological effects; for example, the combination of the question order and response replicability effects [94,95]. The von Neumann measurement theory has a restricted domain of application [67,215].

Quantum instrument formalism is derived from open quantum systems theory through the indirect measurement scheme employing the unitary operator realization of the interaction between a system and a measurement apparatus [114].

## 8. Question Order and Response Replicability Effects and QQ-Equality

The question order effect (QOE) [119] is an effect of the dependence of the sequential joint probability distribution of answers on the questions’ order:

$$p_{AB}\neq p_{BA}.$$

We remark that, for classical probability (see Appendix A),

$$p_{AB}\left(x,y\right)=P\left(\omega \in \Omega :A\left(\omega \right)=x,B\left(\omega \right)=y\right)=\;P\left(\omega \in \Omega :B\left(\omega \right),A\left(\omega \right)=x\right)=p_{BA}\left(y,x\right).$$

Therefore, no-QOE exists in classical probability formalism. However, the experimental statistical data collected in social opinion polls demonstrated QOE [119]. A simple and natural example is the Clinton–Gore opinion poll [119]. In this opinion-polling experiment, people were asked one question at a time, e.g.,

- *A* = “Is Bill Clinton honest and trustworthy?”
- *B* = “Is Al Gore honest and trustworthy?”

Two sequential probability distributions were calculated on the basis of the experimental statistical data, $p_{AB}$ and $p_{BA}$ (first question *A* and then question *B*, and vice versa).

Wang and Busemeyer [120] modelled QOE with observables of the projection type, with state update given by the Lüders projection postulate (see also [121]). In the theory of quantum instruments, these are projective instruments (see Appendix B).

We remark that, for compatible observables of the projection type, [A,B]=0, there is no QOE:

(12) $$Pr\left(A=x,B=y|\psi \right)\equiv \left|\right|E_{B}\left(y\right)E_{A}\left(x\right)\psi {\left|\right|}^{2}=\;\left|\right|E_{A}\left(x\right)E_{B}\left(y\right)\psi {\left|\right|}^{2}\equiv Pr\left(A=x,B=y|\psi \right).$$

Therefore, operators employed in [120] do not commute, and [A,B]≠0. However, this strategy was challenged in [67]. This challenge is related to RRE and Theorem 2 (Section 5).

As was demonstrated in [67], the QOE+RRE combination cannot be modeled by von Neumann observables with a projection state update. In [215], Khrennikov and Basivea tried to resolve this problem by considering a more general class of observables given by so-called atomic quantum instruments, which are the simplest instruments with the state update of the non-projective type (see Appendix B). However, even for such generalized observables, QOE cannot be combined with RRE.

In article [94], Ozawa and Khrennikov overcame this difficulty within quantum instrument theory by using the mathematical construction based on the indirect measurement scheme [114]. The unitary operators describing the interaction of a system and an observable (question) are directly written via their actions. The instruments employed in [94] are non-atomic. In this model, the systems are humans, and the observables are questions asked of them.

Reference [94] contains rather long calculations in Hilbert space. They are not complicated but might be boring to the inexperienced reader. One may be satisfied by the statement that, for QOE+RRE, it is possible to construct quantum instruments by using the indirect measurement scheme. For the more experienced reader, the calculations can serve as the basis for construction of instruments for various combinations of psychological or social effects.

We remark that, in contrast to QOE, which has attracted a lot of attention in experimental psychology and decision-making and which was strongly supported by statistical data [119], RRE is not supported by experimental studies. We can mention just one experiment [170]. We hope that the result and discussion in [161] will stimulate psychologists to perform experiments to check RRE.

Wang and Busemeyer derived the famous QQ-equality (QQE), which is an amazing (and unexpected) property of cognitive and social data [121]. This is a non-parametric inequality which the probabilities of an experiment must satisfy in order for a quantum model to exist for them, as follows:

p(AyBn)+p(AnBy)−p(ByAn)−p(BnAy)=0,

where *A* and *B* correspond to questions with two possible outcomes ‘Yes’ and ‘No’. The joint probabilities are the probabilities of receiving given answers to questions *A* and *B* in the same order as they appear, e.g., P(AyBn) means the probability of obtaining a negative answer to question *B* after obtaining an affirmative answer to question A.

QQE can be easily derived within the standard von Neumann measurement theory, with Hermitian operators and the projection state update. However, von Neumann theory is not powerful enough to describe the combination of QQE with other psychological effects, e.g., QOE+RRE.

Ozawa and Khrennikov [95] proved that the combination QOE+RRE+QQE can be modeled within the theory of quantum instruments.

## Data Availability

Not applicable.

## Appendix A. Classical Probability

Classical probability theory was mathematically formalized, set, and measured in a theoretical framework by Kolmogorov in 1933 [181].

Let Ω be a set of any origin; its points are called elementary events. Consider a collection of subsets F of Ω forming Boolean σ-algebra, i.e., it is closed with respect to countable unions, intersections, and the operation of the complement. If Ω is finite, then F is a collection of all its subsets. Let *P* be a probability measure on F.

The triple P=(Ω,F,P) is called the probability space.

A random variable is mapped as A:Ω→R, having some special property: measurability. We consider only random variables with the discrete range of values. For *A* and its value x, set $\Omega_{A=x}=\left\{s\in \Omega :A\left(s\right)=x\right\}$, and define the probability distribution of *A* as $p_{A}\left(x\right)=P\left(\Omega_{A=x}\right).$ In the discrete case, measurability means that all sets of the form $\Omega_{A=x}$ belong to F.

## Appendix B. A Brief Summary of Quantum Formalism

Let H be a complex Hilbert space; for simplicity, we restrict consideration to finite dimensional spaces. We recall that a *pure quantum state* can be represented by a normalized-by-1 vector of H, i.e., ||ψ||=1. Two vectors that differ only by a phase, i.e., $\psi =e^{i\theta }\phi ,$ represent the same quantum state. When considering a single state, its phase does not play any role, but by manipulating a few states, the relative phases play a crucial role, e.g., in the interference effect.

A density operator ρ is determined by the conditions:

- $\rho =\rho^{★}$ (therefore, it is a Hermitian operator);
- ρ≥0;
- Trρ=1.

The space of density operators is denoted by the symbol D≡D(H).

Density operators represent mixed quantum states, or statistical mixtures of pure states. We note that each pure state ψ can be represented by a density operator, or projection on the state vector, ψ.

The space of linear Hermitian operators in *H* is linear space over real numbers.

We consider linear operators acting in it to be *superoperators*. A superoperator is called positive if it maps the set of positive operators onto itself: for ρ≥0,T(ρ)≥0.

Any map $x\to I_{A}\left(x\right),$ where for each x, the map $I_{A}\left(x\right)$ is a positive super-operator and

(A1) $$\sum_{x}I_{A}\left(x\right):D\to D$$

is called a *quantum instrument.* It represents one of the measurement procedures of an observable A.

The probability of the output A=x is given by the Born rule in the form

(A2) $$P\left(A=x|\rho \right)=\mathrm{Tr}\;[I_{A}\left(x\right)\rho ].$$

We note that measurement with the output A=x generates the state-update by transformation

(A3) $$\rho \to \rho_{x}=\frac{I_{A}\left(x\right)\rho }{TrI_{A}\left(x\right)\rho }.$$

An observable *A* can be measured by a variety of instruments generating the same probability distribution but different state updates.

Let

(A4) $$I_{A}\left(x\right)\rho =P\left(x\right)\rho P\left(x\right),$$

where (P(x)) are projections constrained as

(A5) P(x)P(y)=0,x≠y,

(A6) $$\sum_{x}P\left(x\right)=I.$$

Such an instrument is called a projection instrument. Projection instruments correspond to the standard quantum observables given by Hermitian operators; here, $A=\sum_{x}xP\left(x\right).$ In fact, the later expression is ambiguous: it combines both the Born rule for calculation of probabilities of outcomes and the rule for the quantum state update. The basic idea beyond the quantum instrument theory is to split these two rules and then unify them in superoperators $(I_{A}\left(x\right)).$

The most natural generalization of projective instruments is an atomic instrument. Let (V(x)) be a family of linear operators. Similarly to (A6), the normalization condition has the form

(A7) $$\sum_{x}V{\left(x\right)}^{★}V\left(x\right)=I.$$

An atomic quantum instrument is a super-operator of the form:

(A8) $$\rho \to I\left(x\right)\rho =V\left(x\right)\rho V^{★}\left(x\right).$$

Applications of the quantum instrument theory to quantum information are typically restricted by the use of atomic instruments.

We did not present here essentials of the POVMs theory. See [110] for a brief introduction, and see [6] for applications of open quantum systems and instruments in biology, decision-making and cognition, and social and political sciences.
